# An Unusual Case of Echinococcal Cyst in the Spleen: Diagnostic Challenges and Management Strategies

**DOI:** 10.7759/cureus.81524

**Published:** 2025-03-31

**Authors:** Alhan Samimi, Ashley M Rosander, Lindsay Kadell, Christina Wornom, Benjamin D Brooks

**Affiliations:** 1 Department of Biomedical Sciences, Rocky Vista University College of Osteopathic Medicine, Ivins, USA; 2 Department of Research, Rocky Vista University College of Osteopathic Medicine, Ivins, USA; 3 Department of Biomedical Sciences, Rocky Vista University Montana College of Osteopathic Medicine, Billings, USA; 4 Department of Biomedical Sciences, Rocky Vista University College of Osteopathic Medicine, Ivins, UT, USA

**Keywords:** albendazole, cattle dog, echinococcosis, echinococcus, hydatid cyst, sheep farmer, splenectomy, splenic cyst

## Abstract

Cystic echinococcosis (CE) is a rare parasitic infection characterized by a mass effect within organs depending upon the site of the cyst, with the liver being the most common. Its presentation is usually chronic and may remain asymptomatic for many years. The infection typically presents in patients who live in endemic areas, sheep farmers, or those who work with sheep herding dogs. This presentation in a 26-year-old female living in Salt Lake City, Utah, is rare. This case report serves to highlight a rare presentation of an echinococcal infection in a 26-year-old female who suffered from severe upper gastrointestinal pain, persistent cough, nausea, and vomiting. Diagnostic tests revealed a 13 cm cyst located in her spleen. After confirming the diagnosis via ELISA and Western blot, the cyst tested positive for *Echinococcus*. Despite the potential for cyst rupture and subsequent severe anaphylactic reaction necessitating emergent care, the limited incidence of echinococcal infections within the United States rendered this case a medical enigma with an uncertain diagnosis. This case emphasizes the critical role of biostatistics in diagnosing diseases with low incidence rates.

## Introduction

The tapeworm *Echinococcus* causes cystic and alveolar forms ofechinococcaldisease in humans.*Cystic echinococcosis*(CE) disease is typically caused by an infection by *E. granulosus* in the larval stage. Dogs are the definitive hosts of the tapeworm, and sheep, cattle, goats, and pigs are the intermediate hosts [1]. The cestode resides within the definitive host’s small intestine, where the gravid proglottids release eggs via feces. The intermediate host then ingests these infectious eggs. The eggs hatch and penetrate the host’s small intestine, migrating within the circulatory system to the liver and lungs. Here, they develop into a thick-walled hydatid cyst with the potential to enlarge into multiple daughter cysts. As their life cycle completes, the definitive host becomes infected by ingesting cyst-containing organs from the intermediate host [1].

The overall prevalence of *Echinococcus* is generally underestimated because population surveys are hard to perform in endemic areas. However, due to better diagnostic technology, more cases have been recognized. *Echinococcosis *has become a significant health problem in South America, the Middle East, Sub-Saharan African countries, western China, and the former Soviet Union [2,3,4]. In the United States, local transmission has been observed in California, Arizona, New Mexico, Utah, and Alaska [5]. Risk factors include living in an endemic area where sheep are raised. Transmission occurs through the fecal-oral route via contaminated water and cultivated vegetables in settings where dogs eat the viscera of slaughtered animals [1].

Primary infection with* Echinococcus* is asymptomatic. About half of detected cases are eventually symptomatic, and half are found postmortem [6]. The way in which the infection presents depends on the location and size of the cyst(s) that have formed. Hydatid cysts can form virtually anywhere in the body, with the liver being the most common site, followed by the lungs. They can also be found in the brain, kidneys, spleen, and heart [7]. In the majority of cases, only one organ is affected [6]. The rate at which cysts grow ranges from 1 to 5 centimeters in diameter every year [8]. When the liver is infected, symptoms do not typically occur until the cyst is at least 10 centimeters wide - at which point symptoms such as hepatomegaly, right upper quadrant pain, nausea, or vomiting may occur [6]. Symptoms also occur when greater than 70% of the liver’s volume is engulfed by a cyst [7]. Symptoms are caused by cyst compression of local structures, and cysts in places such as the brain or lungs can lead to life-threatening complications. Patients can remain asymptomatic for about 15 years until the cyst has grown to the point of causing complications or has leaked its contents. 

The most common presentation of symptomatic liver echinococcal infection is pain in the upper abdomen and anorexia. Jaundice can be caused by compression of the bile ducts, and a palpable mass is sometimes present [7]. Cysts in the lungs can present with cough, hemoptysis, or chest pain. In the brain, a myriad of neurological symptoms can occur. Outside of mass effect symptoms, serious allergic reactions can occur if anechinococcal cyst ruptures, which is why puncture of the cyst for diagnostic purposes is contraindicated [7]. 

To diagnose cystic echinococcal infection, the patient’s clinical presentation, serology, and imaging are considered together. Ultrasound is the recommended method of investigation for cysts located in the abdomen as it is both capable of detection and more cost-effective [9]. Computed tomography (CT) and magnetic resonance imaging (MRI) are also used in conjunction to aid in diagnosis. Plain film radiography is unable to detect cysts unless they are calcified and, therefore, on its own, is not a valuable tool in diagnosis [6]. Classification of CE via ultrasound was developed by the World Health Organization Informal Working Groups on *Echinococcus* (WHO-IWGE) in 1995 [10]. Outside of imagining, there are many diagnostic labs that are also used for diagnosis. Detection of *Echinococcus* IgG antibody is more sensitive than *Echinococcus* antigen, and the most frequently used test is EgHF-ELISA to look for IgG in serum [11,12]. 

This case highlights an unusual case of an echinococcal cyst that had engulfed a 26-year-old female’s spleen in Salt Lake City, Utah. The patient initially presented with right upper quadrant abdominal pain, and after ruling out liver and gallbladder pathologies, a 13-centimeter cyst was found incidentally on CT. The patient's treatment course began with many postulated theories and procedures that ultimately led to a complete open splenectomy. Pathology reports from the specimen indicated no echinococcal cystic components, which is why we discuss the importance of biostatistics when it comes to confirming a diagnosis.

## Case presentation

A 26-year-old female presented to the emergency department with severe, sharp epigastric pain that radiated to her flanks bilaterally. The pain began gradually an hour previously after consumption of a fatty meal. She was unable to take a deep inspiration without eliciting pain. She rated the pain a 9/10. She was in the emergency department two days prior for a milder version of the same pain and was diagnosed with constipation. During that visit, she had a right upper quadrant ultrasound, which showed no abnormalities. In addition, she was complaining of a chronic nocturnal cough. 

Upon review of the systems, she endorsed malaise, nausea, cough, and shortness of breath. She denied fevers, chills, night sweats, headaches, vomiting, dysuria, rash, and arthritis. She was having regular bowel movements. 

The patient’s past medical history includes iron-deficiency anemia and exercise-induced asthma. She had no previous abdominal surgeries and no hospitalizations. The only medication she was taking was an oral contraceptive pill. She had no known drug allergies and was up to date on vaccinations. The patient was G0P0 and presented on the second day of her menstrual period. Family history was significant only for breast cancer in a maternal aunt at age 40. 

The patient reported social alcohol use and no history of tobacco or illicit drug use. She worked as a marketing manager and lived with her husband and two dogs (both Shih-Poos) in a townhome in Salt Lake City, Utah. She had traveled to Greece and Mexico two years prior but otherwise had no recent history of travel outside of the United States. 

Upon physical exam in the emergency room, her vitals were blood pressure 129/87, heart rate 104, respiratory rate 20, temperature 98 degrees Fahrenheit, and oxygen saturation 98% on room air. She was in acute distress but did not appear toxic. She had no scleral icterus. She was tachycardic but had regular pulses and normal heart sounds. Her pulmonary effort was normal. Her abdominal exam revealed a soft abdomen with no distention, no rashes or scars, normal bowel sounds, no masses on palpation, right upper quadrant abdominal tenderness, no right lower quadrant tenderness on deep palpation, and voluntary guarding without rebound tenderness. She also had tenderness to percussion of the right costo-vertebral angle. 

The overall assessment of the patient in the emergency room was “A stable, 26-year-old female, who presented with epigastric abdominal pain, RUQ abdominal tenderness and guarding." Labs performed in the emergency room are shown in Table 1. Of note, the patient did not have an elevated white blood cell count, though the eosinophil count in the differential was elevated at 5.9%. Urinalysis revealed trace blood, negative nitrites, negative leukocyte esterase, and negative calcium oxalate crystals. An hCG test was negative. 

**Table 1:** Patient's initial labs in the emergency room

Lab	Patient's value	Result
Hemoglobin	11.3 g/dL (11.6-16 g/dL)	Low
Hematocrit	37.2% (34.0-46.8%)	Normal
Mean corpuscular volume	73.8 fL (79.8-96.9 fL)	Low
Mean corpuscular hemoglobin	22.4 pg (25.1-34.6 pg)	Low
White blood cell count	4.8 K/uL (3.2-10.6 K/uL)	Normal
Neutrophils	2.76 K/uL (1.80-6.80 K/uL)	Normal
Neutrophils %	57.6% (47.0-78.0%)	Normal
Lymphocytes %	32.4% (15.0-39.0 %)	Normal
Eosinophils %	5.9% ( 0.0-5.0%)	High
Aspartate aminotransferase	49 U/L (8-41 U/L)	High
Alanine aminotransferase	39 U/L (10-56 U/L)	Normal
Alkaline phosphatase	77U/L (50-136 U/L)	Normal
Total bilirubin	0.3 mg/dL (0.1-1.2 mg/dL)	Normal
Lipase	33 U/L (12-53 U/L)	Normal

At this point, a cholecystectomy was being considered, given the patient’s presentation similar to that of someone with gallbladder pathology. An ultrasound and HIDA scan were performed, and this showed no gallstones and a normal gallbladder ejection fraction. After ruling out gallbladder pathology, a CT of the abdomen and pelvis with contrast was obtained. This revealed a 12.5 x 12.2 x 13.7 cm cyst encompassing the spleen. Differentials of a splenic mass of this size include splenic cyst, hydatid cyst, splenic pseudocyst, and splenic hemangioma, with the latter being less likely. The patient was admitted, and an ELISA was obtained to rule out* Echinococcus*. Regardless of the assay results, surgical removal with or without interventional radiology (IR) was discussed with the patient. 

Results of the* Echinococcus* IgG antibody ELISA came back positive. The patient was instructed to repeat serology as a false positive is highly likely given her lack of risk factors. Repeat serology resulted as positive; however, cross-reactivity with other parasitic infections was a concern. A Western blot was obtained as it has a higher specificity than an ELISA. This report was again positive for *Echinococcus*. 

**Figure 1:**
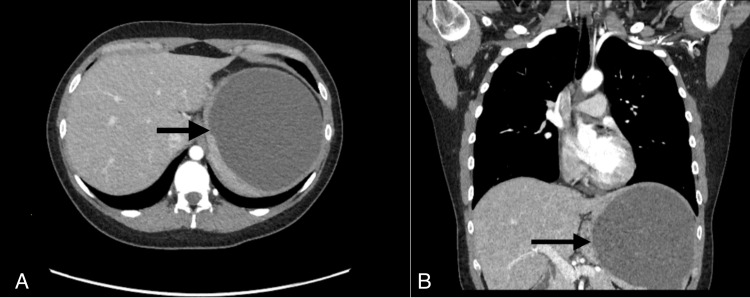
Axial (A) and coronal (B) views of abdominal CT scan showing a large hyper-dense area of a hydatid cyst (black arrows).

A CT of the abdomen and chest was obtained to evaluate for liver and lung pathology, as hepatic and alveolar involvement is common, and the patient was complaining of flank pain and a nocturnal cough. This was negative for cysts in the lung parenchyma however a 14 x 14 cm splenic cyst was detected. CT imaging of the patient's spleen and the hydatid cyst are seen in Figure 1.

**Figure 2:**
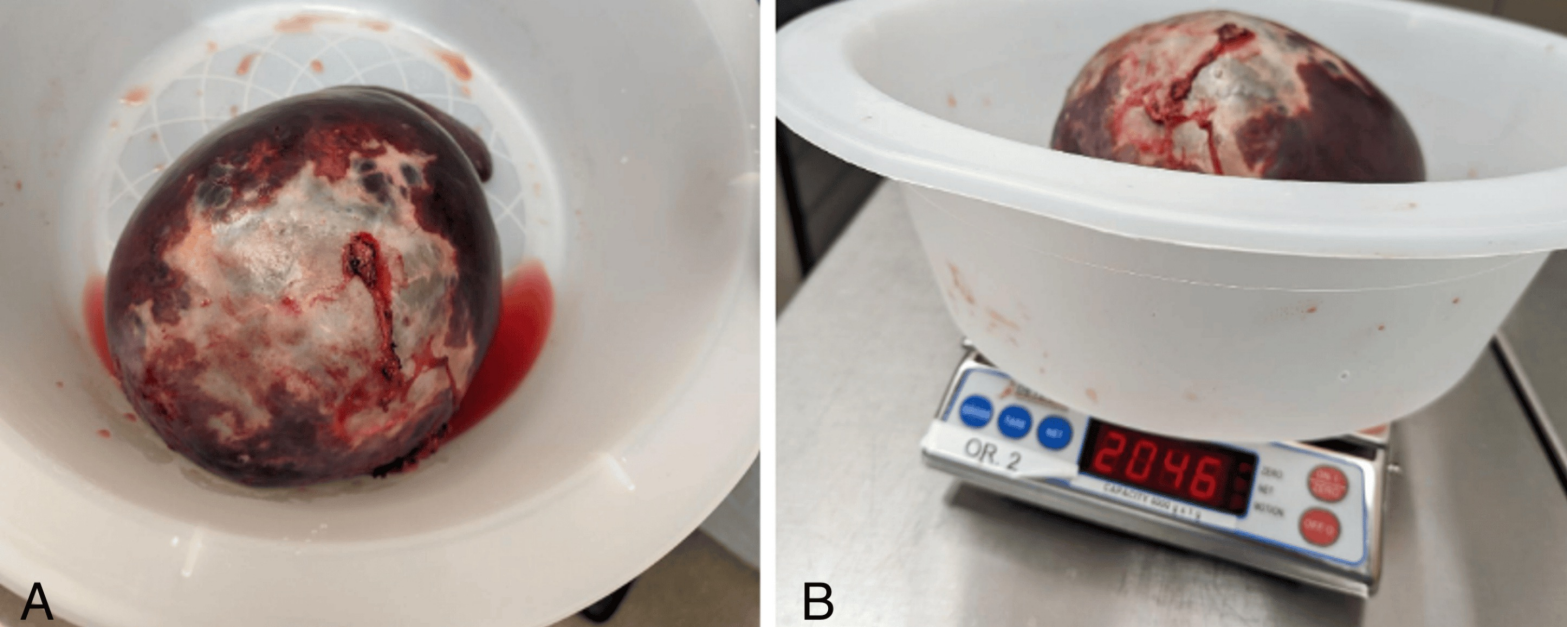
(A) Photographs of the patient’s spleen and the large cyst immediately following splenectomy. (B) The weight of the spleen is shown to be 2,046 grams (4.51 pounds).

Given the size of the patient’s cyst, an open splenectomy was determined to be the best approach. The patient received the typical pre-splenectomy vaccinations (*Haemophilus influenza type B, Streptococcus pneumoniae, Neisseria meningitidis*). To prevent any chance of an anaphylactic reaction, the patient was given 400 mg of albendazole twice daily for one week prior to the open splenectomy and was instructed to continue the medication four weeks post-procedure. In the eight weeks between the initial CT and the splenectomy, the cyst had grown an additional one centimeter in size. Photographs of the patient’s spleen are seen in Figure 2. 

After the splenectomy, the patient recovered in the hospital for four days, and her abdominal pain and shortness of breath completely resolved. During her hospitalization, she was found to have pancytopenia with a low absolute neutrophil count, so her post-surgical albendazole was stopped, as this can be a rare side effect. The final pathology report of the splenic cyst stated, "The clinical history of positive *Echinococcus* antibodies is noted from the electronic medical record. The splenic cyst shows inflammatory features, but no parasites are identified on H&E and polarized light microscopic examinations. Given the positive antibody studies and clinical presentation, the splenic cyst is favored to represent hydatid cyst disease.” 

Two days after discharge, the patient returned to the emergency department with fever, myalgia, and abdominal pain. A CT of the abdomen and pelvis revealed extensive portal vein thrombosis and the patient was started on heparin immediately and re-admitted to the hospital. It was determined that this was a complication of the splenectomy, although it may also have been contributed to by her infectious history with *Echinococcus* and the fact that she was taking oral birth control pills. She was discharged after a few days with a recommendation to continue oral anticoagulation for 12 weeks. 

In summary, this case presentation is of a 26-year-old female who presented to the emergency department with severe right upper quadrant pain and nausea. She was short of breath and tachycardic. An abdominal CT revealed a 13 cm cyst engulfing her spleen. ELISA and Western blot analysis were positive for an echinococcal infection. Notably, this patient lacked common risk factors for splenic hydatid disease, such as recent travel to endemic regions or exposure to livestock, making the diagnosis more unexpected. Ultimately, due to the size and severity of the helminth, the patient was treated with albendazole, an open splenectomy was performed, and her abdominal pain and shortness of breath resolved. 

## Discussion

This report of a 26-year-old female with a splenicechinococcal cyst is rare for several reasons*. Echinococcus* is a rare parasite that predominantly affects those in an endemic area and those with a weakened immune system, usually due to increased age [13]. Given that the patient was born and raised in the United States and is young and generally healthy, the presence of a large splenic cyst weighing five pounds is highly unusual. 

Notably, no parasitic structures were identified in the final pathology report of the splenic cyst, despite positive results from both IgG ELISA and Western blot serologic tests. This discordance between serology and pathology introduces diagnostic uncertainty. While the IgG ELISA is the most widely used test for echinococcal infections, it should be noted that this assay still gives false-negative and false-positive results, so it is necessary to follow this with immunoblot analysis in order to confirm the diagnosis [11]. The ELISA test is not perfect and has a sensitivity ranging between 80% and 90% with a liver hydatid cyst specifically [6]. Similar discrepancies have been reported in the literature, particularly in cases where hydatid cysts are intact and fail to release sufficient antigenic material to provoke a robust immune response detectable on histopathological examination [8]. In addition, serological assays for *E. granulosus* have been shown to cross-react with other parasitic infections, such as *cysticerosis* and *schistosomiasis*, leading to false positives [9,11]. However, it would also have been unusual for this patient to have developed an infection with* E. granulosus *in the first place, considering her lack of exposure to sheep, cattle dogs, and travel outside of the United States. This uncertainty lends itself to a discussion of the positive predictive values of the tests used for the diagnosis of echinococcal infections.

Most of the sensitivity and specificity data available are based on the cyst’s most common locations of the liver and lungs, so there is less certainty around the effectiveness of these assays for a splenic cyst in particular. In addition, it is well understood that the positive predictive value of a test is decreased when the incidence of the disease in question is low in a given population. Sensitivity measures a test’s ability to detect true positives, specificity identifies true negatives, and positive predictive value indicates the likelihood that a positive result reflects the actual condition. In endemic regions, the incidence of CE is around 50 per 100,000 person-years, and the prevalence can be as high as 5-10% in some regions [14]. In the United States specifically, prevalence is likely very low, and a 2017 study revealed just 41 deaths from *Echinococcus *between 1990 and 2007 [15]. Due to the low incidence in the United States, the positive predictive value of the assays performed to identify CE in this patient is in turn decreased as well, making a false positive more likely. Therefore, more studies need to be conducted to further elucidate the validity of testing for* Echinococcus* in less common organs and in non-endemic regions to better serve patients with variable presentations in the future.

## Conclusions

This case reminds medical providers of the vital importance of considering the sensitivity and positive predictive value of all testing, including the gold-standard diagnostic tools, as this patient was diagnosed with a hydatid cyst via imaging and serologic studies and treated as such, but this pathology was not ultimately seen on microscopic examination. In this case, the patient was treated properly via splenectomy as the risk of rupture outweighed conservative treatment in the face of a potential false positive. It is important for physicians to keep a wide differential in mind when using diagnostic tests with imperfections such as those discussed here. In the future, it is important to consider the positive predictive value of a test in a patient with suspected *Echinococcus* in a non-endemic region in the absence of risk factors.

## References

[1] (2024). CDC: symptoms of echinococcosis. https://www.cdc.gov/echinococcosis/symptoms/index.html.

[2] Jenkins DJ, Romig T, Thompson RC (2005). Emergence/re-emergence of Echinococcus spp.--a global update. Int J Parasitol.

[3] Paternoster G, Boo G, Wang C (2020). Epidemic cystic and alveolar echinococcosis in Kyrgyzstan: an analysis of national surveillance data. Lancet Glob Health.

[4] Romig T, Dinkel A, Mackenstedt U (2006). The present situation of echinococcosis in Europe. Parasitol Int.

[5] Moro P, Schantz PM (2006). Cystic echinococcosis in the Americas. Parasitol Int.

[6] Moro P, Reddy DN (2024). Echinococcosis: clinical manifestations and diagnosis. UpToDate.

[7] Wen H, Vuitton L, Tuxun T, Li J, Vuitton DA, Zhang W, McManus DP (2019). Echinococcosis: advances in the 21st century. Clin Microbiol Rev.

[8] Moro P, Schantz PM (2009). Echinococcosis: a review. Int J Infect Dis.

[9] Brunetti E, Kern P, Vuitton DA (2010). Expert consensus for the diagnosis and treatment of cystic and alveolar echinococcosis in humans. Acta Trop.

[10] WHO Informal Working Group (2003). International classification of ultrasound images in cystic echinococcosis for application in clinical and field epidemiological settings. Acta Trop.

[11] Siles-Lucas M, Casulli A, Conraths FJ, Müller N (2017). Laboratory diagnosis of Echinococcus spp. in human patients and infected animals. Adv Parasitol.

[12] McManus DP, Zhang W, Li J, Bartley PB (2003). Echinococcosis. Lancet.

[13] Cohen H, Paolillo E, Bonifacino R (1998). Human cystic echinococcosis in a Uruguayan community: a sonographic, serologic, and epidemiologic study. Am J Trop Med Hyg.

[14] (2024). World Health Organization: echinococcosis. https://www.who.int/news-room/fact-sheets/detail/echinococcosis.

[15] Deplazes P, Rinaldi L, Alvarez Rojas CA (2017). Global distribution of alveolar and cystic echinococcosis. Adv Parasitol.

